# Spirometry use in patients with sickle cell disease with and without asthma and acute chest syndrome: A multicenter study

**DOI:** 10.1002/jha2.42

**Published:** 2020-07-10

**Authors:** Laurie Duckworth, Lucien Vandy Black, Dima Ezmigna, Jeanette Green, Yingwei Yao, Shaun Grannis, Jeff Klann, Reuben Applegate, Gigi Lipori, Tanya Wallace, Diana J. Wilkie

**Affiliations:** ^1^ College of Nursing UF Health University of Florida Gainesville FL; ^2^ Division of Pediatric Hematology and Oncology University of Florida College of Medicine; ^3^ Division of Pulmonology UF Health; ^4^ Biobehavioral Nursing Science AdventHealth; ^5^ Biobehavioral Nursing Science University of Florida College of Nursing; ^6^ Indiana University School of Medicine; ^7^ Harvard Medicine Massachusetts General Hospital; ^8^ University of Texas Health Science Center at Houston, Research; ^9^ UF Health Shands VP Information Services; ^10^ College of Nursing University of Florida; ^11^ Biobehavioral Nursing Science University of Florida College of Nursing

**Keywords:** asthma, pediatric hematology, sickle cell disease

## Abstract

A de‐identified data repository of electronic medical record data, i2b2 (Informatics for Integrating Biology and the Bedside), including four geographically diverse academic medical centers, was queried to determine the use of diagnostic spirometry testing in African American children and young adults 5‐34 years of age with sickle cell disease (SCD) with or without a documented history of asthma and/or acute chest syndrome (ACS). A total of 2749 patients were identified with SCD, of these 577 had asthma and 409 had ACS. Cross‐referencing the CPT code for diagnostic spirometry showed that for patients identified as having SCD, a history of ACS, and a diagnosis of asthma, only 31% across all four centers had spirometry. Having an asthma diagnosis was associated with ACS. Among SCD patients with asthma, the proportion with ACS for the four centers was 47%, 75%, 38%, and 36% respectively. The bivariate association between asthma and ACS for each Center was significant for each (*P* < .001). To summarize, only one third of patients with co‐morbid SCD, ACS, and asthma received the spirometry procedure as recommended in evidence‐based guidelines, suggesting limited testing for changes in pulmonary function. Future studies to determine barriers and facilitators to implementation of pulmonary testing in SCD are warranted.

## 1 INTRODUCTION

The prevalence of diagnostic spirometry testing, an accepted method for asthma screening and monitoring, for children with sickle cell disease (SCD) is unknown. Children with SCD and asthma have a fivefold increased risk for acute chest syndrome (ACS) compared to children with SCD without asthma [1, 2]. Although ACS is a leading cause of mortality in the SCD population and there seems to be increasing recognition of the importance of comorbid asthma and SCD, asthma continues to be underdiagnosed and undertreated in those with SCD [3]. The purpose of this study was to determine the percentage of African American children and young adults of age 5‐34 years with SCD who had spirometry testing done and the correlation with spirometry testing and a diagnosis of asthma and/or ACS.

SCD is a common genetic disorder affecting approximately 100 000 individuals in the United States [4]. According to the Centers for Disease Control and Prevention, approximately 25 million persons in the United States have asthma, a condition influenced by both genetic and environmental factors [5]. A common complication for SCD patients is ACS, characterized by chest pain, tachypnea, fever, respiratory distress, and lung infiltrates [6]. ACS is the leading cause of premature death [7] and the second most common reason for hospitalization in children [8] with SCD. A relationship has been identified among patients comorbid with asthma and SCD, with pediatric patients being five times more likely to experience respiratory symptoms during a vaso‐occlusive episode if asthma is present [9].

The Official American Thoracic Society Workshop Report 2019 identified key unanswered research questions that need to be addressed regarding lung disease in SCD including: what are the features and pathophysiology of lower airway disease in SCD, does lower airway disease impact clinical outcomes in SCD, and is there a role for screening asymptomatic patients with pulmonary function tests (PFTs)? An expert panel with expertise in SCD‐related lung disease concluded that despite improved mortality rates over the past several decades in children and adolescents with SCD, adults with SCD continue to suffer significant morbidity from lung disease and continue to suffer higher mortality [10]. The panel suggests the possibility that informatics and machine learning using large clinical datasets may help identify risk factors and early indicators of ACS. The 2019 American Society of Hematology guidelines as well as the most recently revised National Heart, Lung, and Blood Institute SCD Guidelines, however, do not recommend routine PFTs for children with SCD, unless there are signs or symptoms of respiratory problems by history and/or physical examination [11, 12].

With support of the National Institutes of Health, Clinical and Translational Science Awards, research‐intensive institutions across the nation created Informatics for Integrating Biology and the Bedside (i2b2), software enabling the creation of de‐identified data repositories of electronic medical record (EMR) data. i2b2 offers a unique capability for exploring issues related to the expert panel's unanswered questions [13]. Electronic medical record data from four large, geographically diverse academic medical centers (AMC) were extracted from their i2b2 instances to compare the proportion of pulmonary function testing performed among African American children and young adults 5–34 years old with SCD with and without a history of asthma and/or ACS.

## 2 METHODS

We queried four AMC EMRs using i2b2 for the count of African American patients of age 5‐34 years between 12 January 2010 and 12 January 2015 with SCD. These patients were also evaluated for comorbid diagnoses of asthma and/or ACS. We then performed queries to cross‐reference those patients with the current procedural terminology (CPT) code for spirometry. The i2b2 query included number of patients with (a) SCD, (b) SCD + asthma, (c) SCD + ACS, (d) SCD + ACS + asthma, and (e) SCD + ACS + Asthma + Spirometry. The four academic medical centers were labeled as A, B, C, and D. The ICD‐9 codes queried include asthma 493, SCD 282.6, and ACS 517.3. The Spirometry CPT codes include 94010, 94060, 94070, and 94375. These selected codes were the most widely used at all four centers. The University of Florida Institutional Review Board approved analysis of the de‐identified integrated data repository data.

We utilized the i2b2 data to produce descriptive statistics including frequencies and percentages. Bivariate associations were analyzed using either chi‐square or Fisher's test.

## 3 RESULTS AND DISCUSSION

**FIGURE 1:**
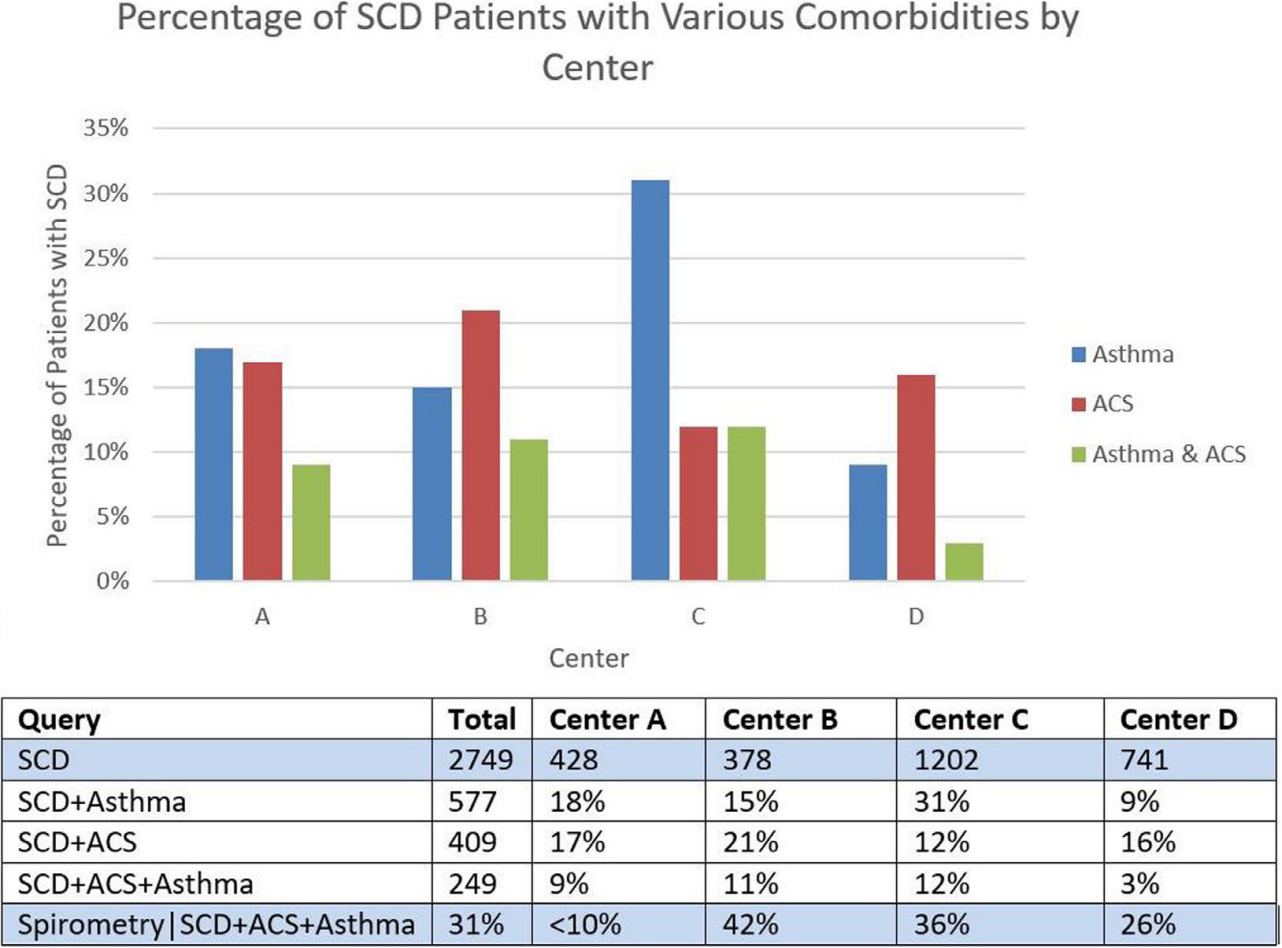
Distributions across four academic health centers: Sickle cell disease, asthma diagnosis, acute chest syndrome diagnosis, and spirometry

As displayed in Figure 1, a total of 2749 African American patients were identified with SCD, of these 577 had asthma and 409 had a history of ACS. Two hundred forty‐nine had SCD, ACS, and asthma, out of which 77 had spirometry performed within the study period. Academic Center C had the highest proportion of SCD patients with asthma at 31%, with A, B, and D having 18%, 15%, and 9% of SCD patients diagnosed with asthma, respectively. The difference in proportions across centers was statistically significant (*P* < .001). On the other hand, the percentage of SCD patients with ACS at the four centers was 17%, 21%, 12%, and 16%, respectively. The between‐center difference was again significant (*P* < .001).

**FIGURE 2:**
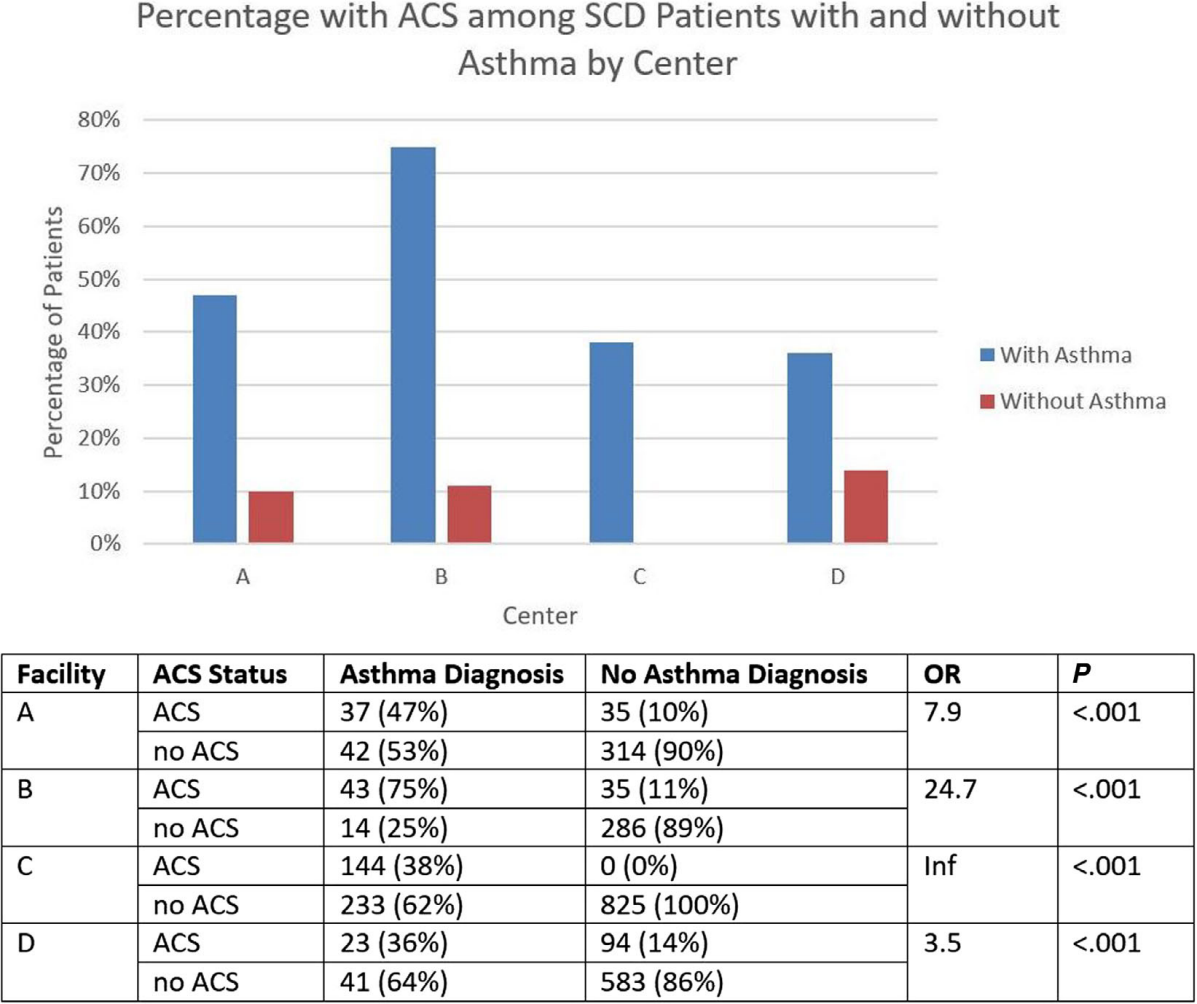
Association of asthma diagnosis and acute chest syndrome diagnosis

A closer look revealed that an asthma diagnosis was associated with ACS (Figure 2). Among SCD patients without asthma, 10‐14% had ACS for Center A, B, and D, whereas 0% had ACS at Center C. On the other hand, among SCD patients with asthma, the proportions with ACS were similar for Centers A, C, and D (47%, 38%, and 36%, respectively) but higher for Center B (75%). Separate analysis of the bivariate association between asthma and ACS for each Center showed that the association was significant for each (*P* < .001). The meaning of these findings is unclear and warrants further study, especially the practices at Center B where those with SCD and asthma had unusually high ACS rate and Center C where those with SCD and no asthma did not have ACS. With regard to odds ratio, the interpretation of which is the outcome and which is the risk factor is based on clinical knowledge. We believe the evidence in the literature shows that asthma is a risk factor for developing ACS.

Notably, for patients identified as having SCD, a history of ACS, and a diagnosis of asthma, only 31% across all four Centers had spirometry (Figure 1). For the same group, Centers B and C had spirometry performed in 42% and 36%, respectively. For unknown reasons, these two Centers, however, had the highest and lowest proportions with ACS.

We appreciate the concern for miscoding raised by reviewers specifically that SCD has a variety of ICD‐9 codes and some patients with ACS may be coded as pneumonia. The data represented were extracted from clinic data and the numbers are consistent with the actual proportion of SCD and ACS patients who are currently being served. One reviewer also expressed that it would be helpful to know the population structure of the SCD patients at each center with regard to age and gender. Again, numbers were consistent with the proportion for age and gender at each center. These data are available to the reader by request. One reviewer was curious as to our selected age range. We were very interested in capturing data on young children and young adults as it is our hope that these patients with asthma are being identified early. We specifically included the 21‐34 age group as we know that during and after transition to adult care practice the morbidity and mortality rate may increase. This could be related to progressive disease; however, we were interested in spirometry testing in this age group. Finally, we appreciate questions related to the study period from 2010 to 2015. We spent a fair amount of time verifying and validating all four centers data. The suggestion to conduct a more current analysis is a great idea, however; one site in particular created a practice change as a result of findings from this study and have developed a combined pulmonology and hematology clinic. It is unknown if any other centers changed practice by virtue of this information.

Despite NHLBI Asthma guidelines recommendations for children with asthma to have spirometry, it appears that only one third of these patients with comorbid SCD, ACS, and asthma received the PFTs [14]. Very few are being routinely tested for abnormalities in pulmonary function or those tests are not being coded and documented appropriately. Better coding and documentation practices for spirometry testing in those with SCD and asthma will allow better access for providers to utilize these test results for asthma management. If the testing is not being done routinely, further research should be done to determine if there are barriers to obtaining appropriate pulmonology evaluation and management.

## AUTHORS CONTRIBUTIONS

VB, DE, JG, and LD identified key elements of the i2b2 query. GL coordinated data retrieval from the four Academic Medical Centers. SG, JK, RA, and LD performed the data queries. YY performed statistical analysis and made figures. TW, YY, DW, and LD analyzed results and wrote the manuscript. All authors reviewed and edited the final manuscript.

## CONFLICT OF INTEREST

The authors declare no conflict of interest.
